# Multi-feature Fusion and Damage Identification of Large Generator Stator Insulation Based on Lamb Wave Detection and SVM Method

**DOI:** 10.3390/s19173733

**Published:** 2019-08-29

**Authors:** Ruihua Li, Haojie Gu, Bo Hu, Zhifeng She

**Affiliations:** Department of Electrical Engineering, Tongji University, Shanghai 201804, China

**Keywords:** stator insulation, multi-feature, support vector machine, damage identification

## Abstract

Due to the merits of Lamb wave to Structural Health Monitoring (SHM) of composite, the Lamb wave-based damage detection and identification technology show a potential solution for the insulation condition evaluation of large generator stator. This was performed in order to overcome the problem that it is difficult to effectively identify the stator insulation damage the using single feature of Lamb wave. In this paper, a damage identification method of stator insulation based on Lamb wave multi-feature fusion is presented. Firstly, the different damage features were extracted from time domain, frequency domain, and fractal dimension of lamb wave signals, respectively. The features of Lamb wave signals were extracted by Hilbert transform (HT), power spectral density (PSD), fast Fourier transform (FFT), and wavelet fractal dimension (WFD). Then, a machine learning method based on support vector machine (SVM) was used to fuse and reconstruct the multi-features of Lamb wave and furtherly identify damage type of stator insulation. Finally, the effect of typical stator insulation damage identification is verified by simulation and experiment.

## 1. Introduction

Stator insulation of large generators has a laminated composite structure. In long-term service, stator insulation is exposed to a combination of electrical, thermal, and mechanical stresses. They gradually cause the aging of insulation structure, resulting in internal or surface insulation damages, such as void, delamination, puncture crack, and surface crack, etc. These damages eventually can lead to failure and breakdown of large generator stator insulation [1,2,3,4]. The occurrence of such accidents not only endangers the generator itself, but also results in the power failure of the whole system, which causes huge economic losses. Therefore, timely and accurate detection and identification of stator insulation structure damage can provide effective and reliable reference information for insulation status diagnosis and life assessment of large generator stator.

Implement of condition monitoring (CM) on stator insulation holds the promise of enhancing the safety and economic operation of a large generator. Structural Health Monitoring (SHM) is a promising method for the non-destructive defect detection of composites [5]. Its purpose is to detect the damage occurring inside or on the surface of the structure in real time, so as to carry out early diagnosis and prediction and prevent the accidents before it happens. In addition, SHM can reduce the maintenance cycle and cost, and ultimately achieve the purpose of avoiding catastrophic accidents. Lamb wave is a guided wave which propagates in thin plate structures. It has been developed as a new non-destructive testing technology for effectively monitoring the health of structures due to its easy detection of minor damage in structures. Considering the potential benefits of Lamb waves in defect detection of laminated composite structure [6], some progress has been made in the study of stator insulation damage detection. Lamb wave can not only detect the damage of stator insulation, but also locate the damage and even conduct damage imaging [7,8,9]. Thus, Lamb wave has good potential application value in health monitoring of large generator stator insulation.

The type of stator insulation damage can reflect the state of stator insulation aging to a certain extent. So the degree of stator insulation aging can be evaluated by identifying typical stator insulation damage categories. Due to the multi-mode and dispersion of Lamb wave, it is difficult to explain the damage detection signal, which seriously affects the effect of damage identification. In addition, the stator insulation of large motor is a composite material structure, and the Lamb wave propagation process is very complex, the reflection and scattering of Lamb wave make it difficult to extract the damage features, thus causing great obstacles to the identification of damage types. In particular, using a single damage feature for damage identification can also reduce the accuracy of stator insulation damage type identification. Moreover, the traditional damage classification method often depends on manual intervention and experience, and the results of damage identification are quite different. With the rapid development of computer technology, especially machine learning algorithms, intelligent damage identification has become an inevitable trend. At present, among the common machine learning algorithms, clustering algorithm (CL) [10] classifies samples according to the strong similarity of sample features, but there are still some problems such as poor stability and sensitivity of initial clustering centers. Artificial Neural network (ANN) [11] is a computer learning network system established by imitating the topology of human brain neurons, which can solve complex non-linear mapping problems. However, the ANN algorithm has some shortcomings, such as slow convergence speed and large sample demand. Due to the difficulty in obtaining insulation damage samples of large generator stator, the number of damaged samples is small, which limits the application of neural network and clustering algorithm. As a machine learning algorithm based on statistical learning and structural risk minimization, Support Vector Machine (SVM) [12,13] can solve small sample, no-linear and high dimensional pattern recognition problems, which has been applied in the field of damage identification based on Lamb wave signals [14]. In this paper, a stator insulation damage identification method based on Lamb wave multi-feature fusion is proposed. Hilbert transform (HT), power spectral density (PSD) analysis, fast Fourier transform (FFT) and wavelet fractal dimension (WFD) methods were used to extract features of stator insulation damage detection signal respectively. Based on these damage features, a damage feature fusion algorithm and an SVM classification algorithm was used to identify the types of stator insulation damage. 

This paper is organized as follows. In Section 2, the principle of stator insulation damage identification based on Lamb wave is described. Section 3 introduces the methods of stator insulation damage feature extraction from time domain, frequency domain, and fractal dimension respectively and multi-feature fusion. In Section 4, the design of SVM classifier is introduced. The finite element simulation is used to analyze the insulation damage identification of large motor stator based on Lamb wave multi-feature fusion in Section 5. In Section 6, the validity and accuracy of this method is verified through experiments. Finally, conclusions are drawn in Section 7.

## 2. Principle of Stator Insulation Damage Identification Based on Lamb Wave

**Figure 1:**
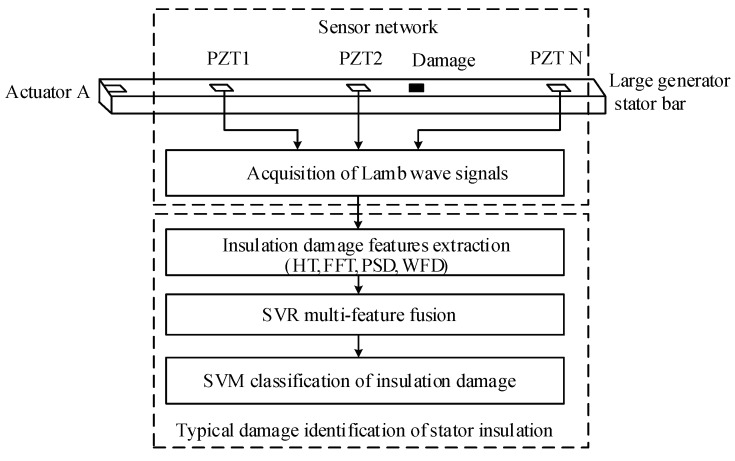
Schematic diagram of stator insulation damage identification using Lamb wave.

Lamb wave is a kind of guided wave propagating in plate structure. When there is damage in stator insulation structure, Lamb wave will reflect and scatter with the damage, which will have different effects on Lamb wave signal. By analyzing Lamb wave signal, the existence and degree of insulation damage can be detected. The principle of damage identification based on Lamb wave is shown in Figure 1. Actuator A excites Lamb wave in generator stator insulation structure, and other PZTs receive Lamb wave detection signals of stator insulation damage. Then different damage features are extracted based on the damage detection signals and multi-feature fusion is carried out. Finally, the accurate identification of stator insulation damage is realized by machine learning classification algorithm.

## 3. Extraction and Fusion Methods of Stator Insulation Damage Features

The feature extraction of stator insulation damage is an important part for damage identification. In this paper, the stator insulation damage features are extracted from time domain, frequency domain and fractal dimension respectively. These methods are described as follows.

### 3.1. Damage Feature Extraction Based on Time Domain

The energy distribution characteristic of Lamb waves signals can be obtained by Hilbert transform [15,16]. The time domain feature can characterize the stator insulation damage condition to a certain extent. For Lamb wave signal *x*(*t*) in time domain, its HT definition is shown by the following [17]:

(1) $$H(t)=\frac{1}{\pi }\int_{+\infty }^{-\infty }\frac{x(t)}{t-\pi }d\pi$$

The envelope of Hilbert transform can represent the energy distribution feature of the signalin time domain, and the damage characteristic of stator insulation can be reflected by extracting the amplitude of the envelope. In order to obtain the envelope of Lamb wave signal in time domain. The analytic signal can be expressed as:

(2) $$Z(t)=x(t)+jH(t)=e(t)e^{i\phi (t)}$$

where, Lamb wave signal x(t) acts as the real part and Hilbert transform signal H(t) acts as the imaginary part. According to Equation (2), the envelope is $e(t)=\sqrt{x^{2}(t)+H^{2}(t)}$, and the phase is ϕ(t)=arctan(H(t)/x(t)). By this way, the amplitude distribution characteristics of the Lamb wave signal in the time domain can be gained.

### 3.2. Damage Feature Extraction Based on Frequency Domain

By extracting frequency components and amplitude characteristics of lamb wave signal in frequency domain, which can be used for identifying the damage of stator insulation. Based on frequency domain analysis theory, the Fast Fourier transform (FFT) [18,19] and power spectral density (PSD) [20] analysis are used to extract the frequency domain features of Lamb wave signals in this paper. FFT algorithm is developed from Discrete Fourier Transform (DFT). Compared with DFT, the operation times of FFT are greatly reduced, and the operation efficiency is improved. The DFT of a discrete Lamb wave signal x(n),n=0,1,…,N−1 is expressed as

(3) $$X(k)=\sum_{n=0}^{N-1}x(n)W_{N}^{kn}k=0,1,\ldots ,N-1,W_{N}=e^{-j\frac{2\pi }{N}}$$

According to the symmetry and periodicity of $W_{N}$, the N-point DFT is decomposed into two N/2-point DFTs. The discrete Lamb wave signal x(n) is decomposed into the sum of even and odd sequences, which are both N/2 points:

(4) $$x(n)=x_{1}(n)+x_{2}(n)$$

where, $x_{1}(n)$ is an even sequence of Lamb wave signal and $x_{2}(n)$ is an odd sequence of Lamb wave signal. Due to $W_{N}^{2kn}=e^{-j\frac{2\pi }{N}2kn}=e^{-j\frac{2\pi }{N/2}kn}=W_{N/2}^{kn}$, the formula (3) can be shown as

(5) $$\begin{array}{ll} X(k) & =\sum_{n=0}^{\frac{N}{2}-1}x_{1}(n)W_{N}^{2kn}+\sum_{n=0}^{\frac{N}{2}}x_{2}(n)W_{N}^{(2n+1)k}\\ & \;=\sum_{n=0}^{\frac{N}{2}-1}x_{1}(n)W_{N/2}^{kn}+W_{N}^{k}\sum_{n=0}^{\frac{N}{2}-1}x_{2}(n)W_{N/2}^{kn}\;,\;k=0,1,\ldots ,N-1\\ & \;=X_{1}(k)+W_{N}^{k}X_{2}(k) \end{array}$$

where, $X_{1}(k)$ and $X_{2}(k)$ are the DFTs of $x_{1}(n)$ and $x_{2}(n)$ respectively. By repeating the iteration of Equation (5), the amplitude features of Lamb wave signal in frequency domain can be extracted by FFT transform. 

The results of previous research [21] indicated that the peak power spectrum of Lamb wave signal varies with the change of damage size. Therefore, PSD peak feature parameter can characterize stator insulation aging characteristics to a certain extent. In order to simplify the calculation, the Lamb wave signal is approximated as a weak stationary signal for correlation processing in this study. According to Reference [22], its definition is:

(6) $$\left\{ \begin{array}{l} S_{X}(\omega )=\int_{-\infty }^{+\infty }R_{X}(\tau )e^{-i\omega \tau }d\tau \\ R_{X}(\tau )=\frac{1}{2\pi }\int_{-\infty }^{+\infty }S_{X}(\omega )e^{i\omega \tau }d\omega \end{array}\right.$$

where, $R_{X}(\tau )$ is the autocorrelation function of Lamb wave signal, the Lamb wave energy characteristic can be obtained by calculating $S_{X}(\omega )$, which can be used for identifying the damage of stator insulation. 

### 3.3. Damage Feature Extraction Based on Fractal Dimension

Due to the fractal dimension can quantitatively characterize the irregularity and self-similarity features of signals [23], which has been applied for structural health monitoring based on Lamb wave detection [24]. A wavelet fractal dimension (WFD) method is used to obtain the features of Lamb wave signal in this study.

Firstly, the range of each frequency band is determined by wavelet decomposition, the Lamb wave signal is decomposed by wavelet transform. The frequency range of detail component $d_{2^{j}}$ of each layer can be obtained as follows:

(7) $$\frac{f_{s}}{2^{j+1}}<\omega <\frac{f_{s}}{2^{j}}$$

where, $f_{s}$ is the sampling frequency, j is the number of decomposition layers, ω is the frequency of each component. On this basis, the characteristics of each frequency component near the center frequency of Lamb wave can be analyzed furtherly. The variance of each component is obtained as:

(8) $$Var(d_{2^{j}})=\frac{1}{N_{j}-1}{\sum \left(d_{2^{j}}-m_{d_{2^{j}}}\right)}^{2}$$

where, $m_{d_{2^{j}}}$ is the mean of the frequency component $d_{2^{j}}$ at scale j, and $N_{j}$ is the sample points of the Lamb wave signal. Then, the slope β of the line can be obtained. Finally, the fractal dimension *D* can be obtained as:

(9) $$D=\frac{5-\beta }{2}$$

where, β is the slope of the line fitted, which can be gained by fitting the relationship between the logarithm of $Var(d_{2^{j}})$ and scale j.

Based on the extraction of damage characteristics, multi-feature fusion and damage identification of stator insulation can be further carried out.

### 3.4. Multi-feature Fusion of Stator Insulation Damage

Damage classification method of stator insulation based on single feature can result in the problem of low rate of damage identification. Multi-feature fusion identification is a comprehensive processing of target multi-feature information, which can obtain more accurate and comprehensive estimation and judgment than single feature. In order to identify the type of stator insulation damage effectively, Hilbert transform (HT), power spectral density (PSD) analysis, fast Fourier transform (FFT) and wavelet fractal dimension (WFD) methods were respectively used to extract features of Lamb wave signals received in stator insulation damage detection. In addition, since these damage features are directly related to the geometric dimension of insulation damage, the effect of stator insulation damage identification can be further improved if the geometric dimension of insulation damage and the extracted multi-features are comprehensively considered. So support vector regression (SVR) algorithm is used to predict the damage size based on the extracted damage features in this paper, then the geometric dimension and the extracted features of stator insulation damage are fused to further identify the type of stator insulation damage.

## 4. Classifier Design of Stator Insulation Damage Identification

The design of classifier is an important part of insulation damage identification. In practical application, it is very difficult to obtain sufficient typical damage samples of stator insulation. Therefore, in the case of small samples, the design of classifier is very critical, which directly determines the effect of stator insulation damage identification. Because SVM has obvious advantages in solving small sample, non-linear and high-dimensional pattern recognition problems [25] and has good generalization ability. Thus, in this study, an SVM machine learning method is used to design the classifier of identifying stator insulation damage.

The classical support vector machine (SVM) is mainly designed for binary classification problem, and its design principle is as follows. It is assumed that the given training data sample set is $\left(x_{i},y_{i}),i=1,2,\ldots ,n,x\in R^{d},y_{i}\in \{1,-1\right\}$, where *n* is the number of training samples, *d* is the dimension of each training sample, and $y_{i}$ is the category number. At first, a non-linear function φ is used to map the sample data from the original space to the N-dimensional feature space:$\varphi (x)=(\varphi_{1}(x),\varphi_{2}(x),\ldots ,\varphi_{N}(x))$. Then, a separating hyperplane is constructed in the feature space, as follows [26]:

(10) y(x)=sign(ω·φ(x)+b)

where ω is the weight parameter of separating hyperplane and *b* is the scalar threshold. Lagrange principle is used to solve the optimization problem and the optimal separating hyperplane is obtained as:

(11) $$\sum_{i=1}^{M}\alpha_{i}y_{i}K(x_{i},x)+b=0$$

where $\alpha_{i}$ is the Lagrange’s multiplier, $K(x_{i},x)=\varphi (x_{i})\cdot \varphi (x)$ is the kernel function, and *M* is the number of support vectors. The general form of SVM classifier can be expressed as:

(12) $$f(x)=\mathrm{sign}\{\sum_{i=1}^{M}\alpha_{i}y_{i}K(x_{i},x)+b\}$$

where sign{} is a symbolic function, and the category of *x* can be determined by the positive or negative of classifier f(x).

**Figure 2:**
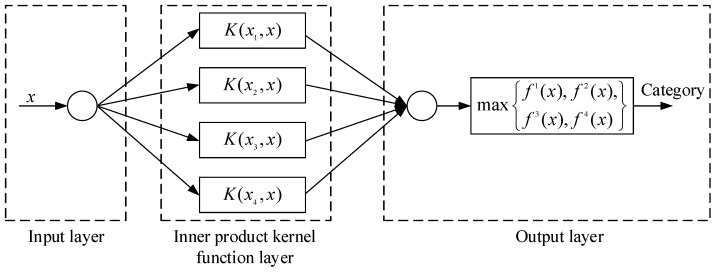
Structure of Support Vector Machine (SVM) classifier.

Various types of insulation damage can be gradually formed in the aging process of large generator stator insulation. Stator insulation damage identification is essentially a multi-classification problem. Therefore, a multiclass SVM method is used to identify the types of stator insulation damage in this paper, and a multiclass SVM problem can be converted into several binary SVM problems [27,28]. The training sample set of stator insulation damage types is given as $\left(x_{i},y_{i}),i=1,2,\ldots ,n,x\in R^{d},y_{i}\in \{1,2,\ldots ,N\right\}$, where *n* is the number of training samples, *d* is the dimension of each training sample, and *N* is the category of stator insulation damage. The classification output function of the *k*th ($k\in y_{i}$) classifier can be established as:

(13) $$f^{k}(x)=\sum_{i=1}^{M}\alpha_{i}^{k}y_{i}^{k}K(x_{i},x)+b^{k}$$

where *x* is the testing sample, and *M* is the number of support vectors. If $f^{k}(x)$ is positive, the testing sample *x* is classified in class *k*; otherwise, the testing sample *x* is classified other classes. Its structure is shown in Figure 2.

As can be seen from Figure 2, the SVM classifier is similar to a three-layer feedforward network. The first layer is the input layer, and the number of nodes in the input layer is the number of insulation damage samples (Figure 2 takes only one input sample as an example). The second layer is the inner product kernel function layer. According to [29], radial basis kernel function has strong applicability for sample size and dimension. Therefore, basis kernel function is used in SVM classifier design in this paper. The third layer is the output layer, which directly outputs the insulation damage category of the samples. Since different damage features have different parameter dimensions, in practical applications, the samples data is firstly normalized. The normalization method is as follows:

For the sample data x(n),n=1,2,…,N, the average value of the sample can be obtained:

(14) $$\bar{x}=\frac{1}{N}\sum_{i=1}^{N}x(i)$$

Then the normalized value can be obtained:

(15) $$y(k)=\frac{x(k)}{\bar{x}},k=1,2,\ldots ,N$$

where, y(k) is the normalized value of the sample data.

**Figure 3:**
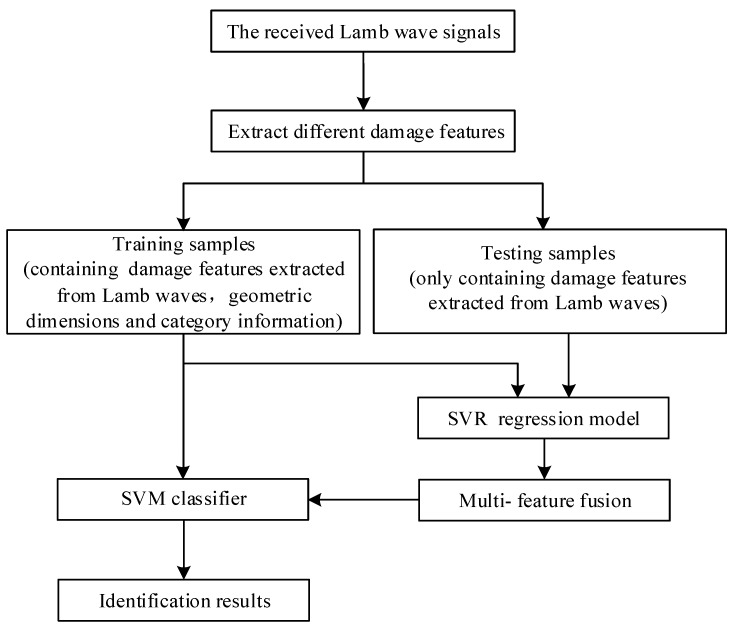
Procedure of stator insulation damage identification based on SVM.

The procedure of stator insulation damage multi-feature fusion and damage identification based on lamb wave detection and SVM method is shown in Figure 3.

Based on the damage feature extraction methods of stator insulation above, the damage features based on Lamb wave detection signals are extracted respectively. Combining the geometric dimensions, the extracted damage features and categories information, the SVM training samples are constructed and the SVM classifier is trained. Because the identification of stator insulation damage types can be further improved by the combination of damage geometric dimension and the extracted damage features. In this study, the support vector regression (SVR) is used to fuse the damage geometric dimensions and the extracted damage features. Combining the geometric dimension information of stator insulation damage and the extracted damage features information, the SVR training samples are constructed and the SVR regression model is trained. Finally, by fusing the damage features with the regression predicted geometric dimensions, the categories of the stator insulation typical damage testing samples can be identified effectively.

## 5. Numerical Simulation and Result Analysis of Stator Insulation Damage Identification

In this study, the finite element method (FEM) software ABAQUS is used to establish the stator insulation damage model, and the effectiveness of the stator insulation damage identification method based on Lamb wave multi-feature fusion is verified by simulation analysis data.

### 5.1. Stator Insulation Structure Model

**Figure 4:**
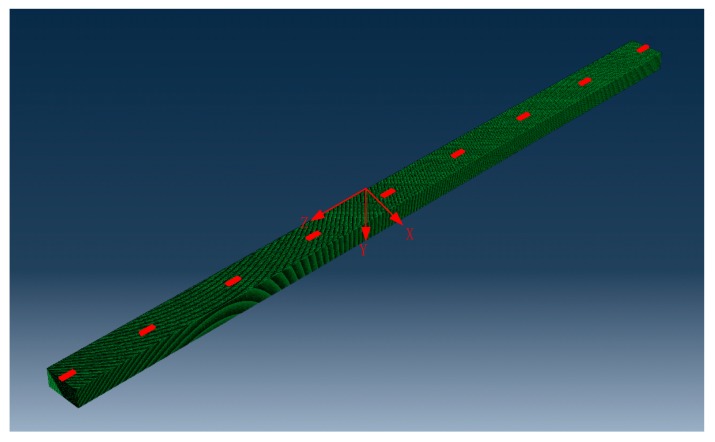
Finite element model of stator insulation structure.

**Figure 5:**
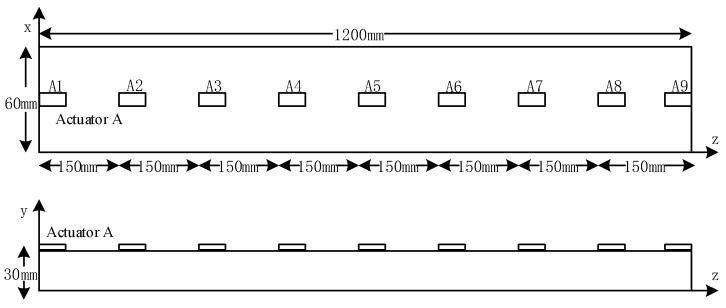
Map of sensor network configuration.

The three-dimensional FEM model of the stator bar is established with geometry of 60 mm in width (x direction), 30 mm in height (y direction) and 1200 mm in length (z direction), as shown in Figure 4. Element size and time step are two important parameters in finite element simulation. In this paper, the element is set as a cube with a side length of 1 mm and the time step is set to 0.1 us. The material parameters of the stator insulation were tested as follows [30]: Young’s modulus of 35 GPa, density of 1720 kg/m^3^, and Poisson’s ratio of 0.2. Moreover, PZT wafers are surface-attached on the insulation of stator bar using an adhesive epoxy to establish the active sensor network. PZT wafers are configured in the middle of widths (x direction) and 150 mm apart in z direction, as illustrated in Figure 5. PZT A1 is served as actuator to excite Lamb waves, and other PZT wafers act as sensors to acquire the Lamb wave signals. According to the material parameters of the stator insulation tested in [30], the dispersion curve of lamb wave propagation can be obtained. According to the previous research [7,9,31], the consensus rules to choose a proper frequency from dispersive cure are that: minimize the propagation modes, large discriminability of group velocity to separate each individual wave mode and stabilized group velocity. Therefore, in this study, the excitation signal adopts a five-cycle Hanning-windowed sinusoidal tone-burst with a central frequency of 13 kHz.

**Figure 6:**
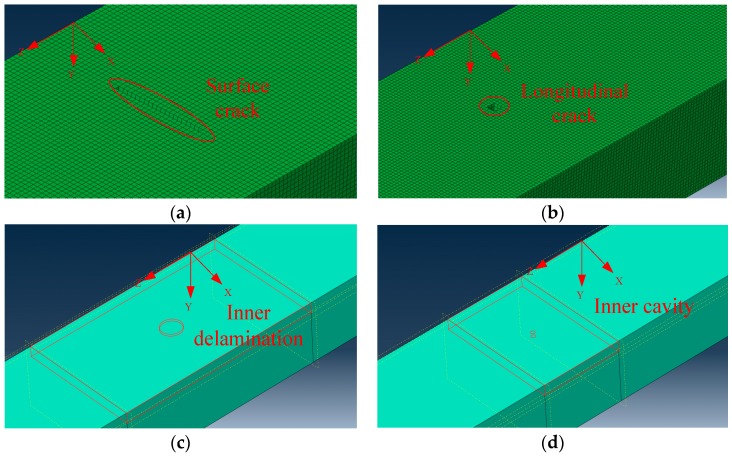
Typical Damage Models of Stator Insulation. (**a**)Surface crack; (**b**) Longitudinal crack; (**c**) Inner delamination; (**d**) Inner cavity.

The typical stator insulation damage of large generator mainly includes four kinds, i.e., surface crack, longitudinal crack, inner delamination, and inner cavity. From the perspective of industrial application, insulation damage may occur at any location of stator bar. In this paper, a PZT sensor network is constructed to realize excitation and reception of Lamb, so as to acquire insulation damage detection signals for stator insulation damage identification. In PZT sensor networks, any PZT can either excite Lamb waves or receive Lamb waves. Therefore, in practical applications, there are multiple paths for Lamb wave excitation and reception (one PZT excites Lamb wave, another PZT receives Lamb wave, which can be called one path or mode of operation). In this way, the possible damage at any location can be detected through sensor networks. In this study, only one example is given to illustrate the relationship between excitation and reception. The working modes of all paths are similar. Figure 6 exhibits the numerical simulation models of the four kinds of damage, which are simulated and configured at the same place (1120 mm in z-direction), and models of different damage sizes are established.

### 5.2. Analysis of Insulation Damage Identification Results Based on a Single Feature

**Figure 7:**
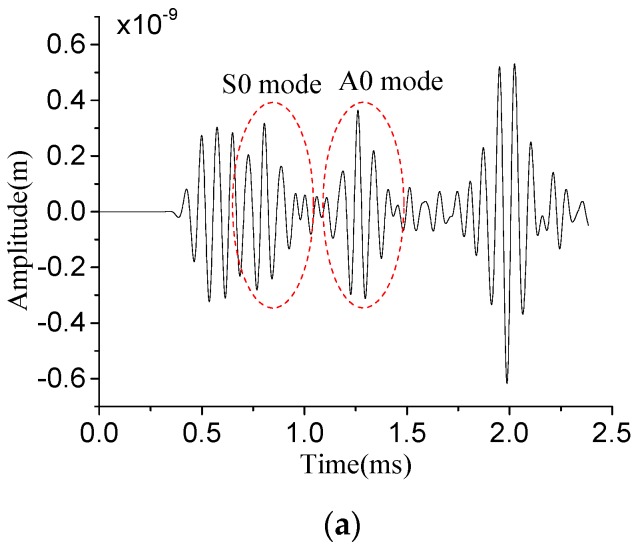

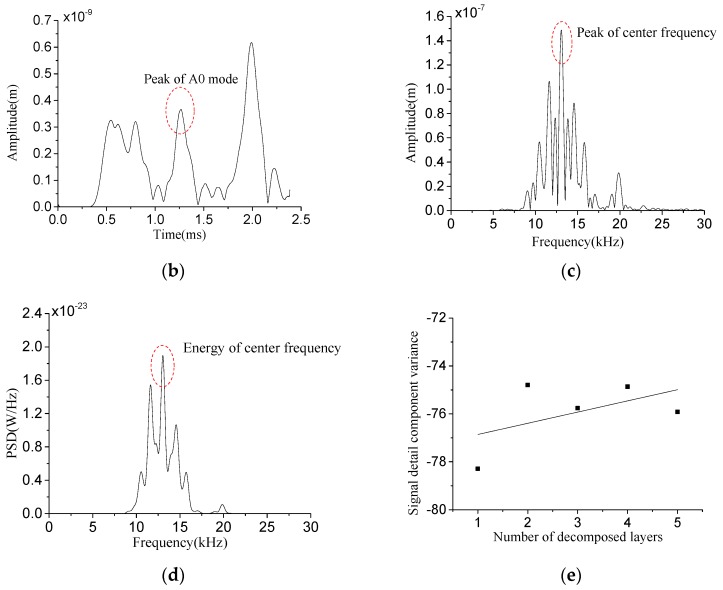
Lamb wave feature extraction of surface crack. (**a**) Received Lamb wave signal; (**b**) Hilbert wave packet of Lamb wave signal; (**c**) fast Fourier transform (FFT) amplitude of Lamb wave signal; (**d**) power spectral density (PSD) energy of Lamb wave signal; (**e**) Fitting straight line of Lamb wave wavelet fractal.

According to the Section 5.1, one non-damage model is established firstly, then other twenty-eight typical damage models of stator insulation are established, including seven surface crack models, nine longitudinal crack models, nine inner delamination models and three inner cavity models. The features of Lamb wave signals obtained from different damage sizes are extracted by HT, FFT, PSD and WFD, respectively. In this study, the PZT sensor network was constructed to realize the excitation and reception of lamb waves. So as to obtain the insulation damage detection signal for stator insulation damage identification. The same damage features are firstly extracted from Lamb wave detection signals collected by each receiving sensor. Then the extracted damage features are used for the fusion of multi-features and the identification of typical damage. Taking the inner delamination (15 mm (x direction) × 2 mm (y direction) × 15 mm (z direction)) as an example, PZT A1 is served as actuator to excite Lamb wave, and PZT A9 acts as sensor to acquire the Lamb wave signal. Different features extraction results are shown in Figure 7.

According to [7,9,30], when the excitation frequency is about 13 kHz, the Lamb wave has the least modes (including only the S0 and A0 wave modes). In addition, the A0 mode wave packet amplitude has a smaller attenuation than the S0 mode wave packet amplitude. A0 mode wave packet can effectively detect stator insulation damage, thus the Hilbert amplitude of A0 mode wave packet is extracted. Since the center frequency of Lamb wave is 13 kHz, the FFT amplitude and PSD energy at this frequency are extracted. The wavelet fractal dimension is obtained according to Equation (8). The feature extraction results of stator insulation damage are shown in Table 1.

As shown in Table 1, the four kinds of damage features vary with the damage size, which indicates that these features can effectively reflect the stator insulation damage. In order to identify the type of stator insulation damage, one non-damage sample, three surface crack samples, five longitudinal crack samples, five inner delamination samples and two inner cavity samples are randomly selected as training samples, and the remaining samples are used as testing samples in the SVM classifier. To compare the identification effect using SVM classifier, three-layer BP neural network classifier is used to identify insulation damage. The identification results are shown in Table 2.

It is seen from Table 2 that the accuracy of damage identification based on single feature is relatively low. For the same feature, the classification effect of SVM classifier is better than that of ANN classifier.

### 5.3. Analysis of Insulation Damage Identification Results Based on Multi-feature Fusion

To overcome the problem of low identification rate of single feature, the type of stator insulation damage is identified based on multi-feature fusion. According to the four damage features, the insulation damage sizes $\overset{\wedge }{x},\overset{\wedge }{y},\overset{\wedge }{z}$ are predicted by SVR. 

In order to evaluate the quality of the regression model predicting results, a relative error evaluation method is used to this issue in this paper. The relative error calculation formula is as follows:

(16) $$\left|\delta \right|=\frac{\Delta }{L}\times 100\%$$

where Δ is the absolute error, that is, the difference between the predicted value and the actual value; L is the actual value. Taking 13 testing samples as an example, their predicted results are evaluated, and the results are shown in Table 3.

According to Table 3, it can be seen that the absolute value of the relative error of the SVR prediction results is less than 10%, which indicates that the method can accurately predict the geometrical dimensions of the stator insulation damage.

The predicted insulation damage sizes are fused with the damage features to identify the insulation damage. Compared with the identification effect based on damage features of Lamb wave, the results are shown in Table 4.

Table 4 indicates that the accuracy of insulation damage identification using multi-feature is better than that of single feature. In addition, the accuracy of damage identification can be further improved by predicting the damage geometry size of the testing samples and fusing it with the damage features of Lamb wave, indicating a good capability to identify the type of the stator insulation damage. With the increase of training samples, the accuracy of damage identification is improved. Moreover, the damage identification accuracy of SVM classifier is also better than that of ANN classifier under the same feature quantities.

## 6. Experiment and Result Analysis of Stator Insulation Damage Identification

**Figure 8:**
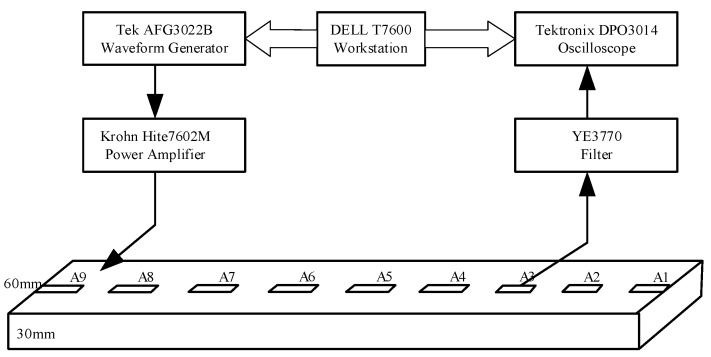
Experiment system of stator insulation damage detection.

To verify the damage identification effect of large generator stator insulation based on Lamb wave multi-feature fusion, a damage identification experiment is performed. The experimental system is set up, as shown in Figure 8.

In the system, an arbitrary waveform generator (AFG3022B) is used to generate Lamb wave excitation waveforms. A power amplifier (7602M) is used to drive PZT actuator. An oscilloscope (DPO3014) can acquire Lamb wave signals. DELL T7600 workstation is used for signal processing. PZT wafers are attached to the surface of the stator insulation. PZT A9 acts as an actuator to excite the Lamb wave signal, and PZT A1~A8 act as sensors to receive the Lamb wave propagation signals. In the experiment, the arbitrary waveform generator generates a preset excitation signal to PZT A9, and PZT A1~A8 receive the Lamb wave signals which can be collected and saved by oscilloscope for further analysis and processing.

**Figure 9:**
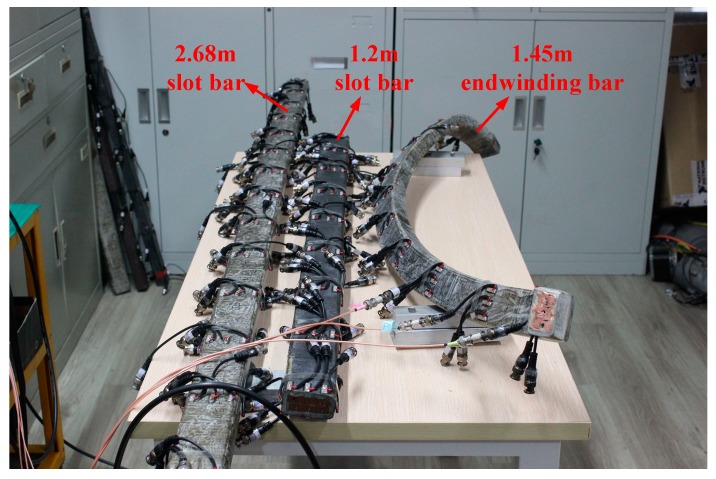
Specimen of stator bars in damage detection experiments.

**Figure 10:**
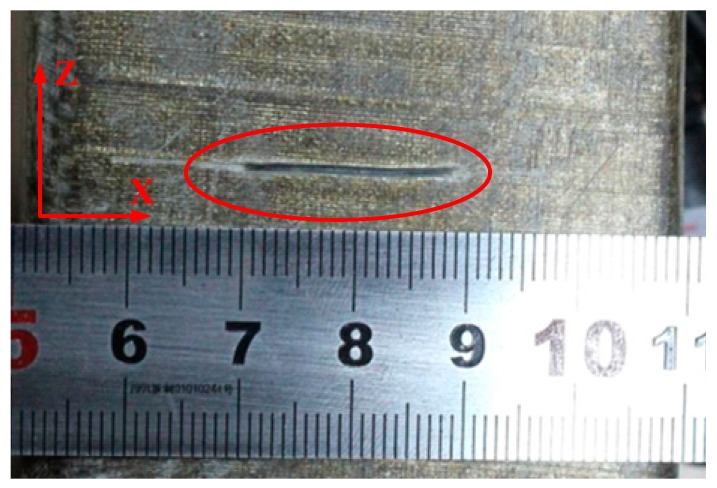
The surface crack damage.

**Figure 11:**
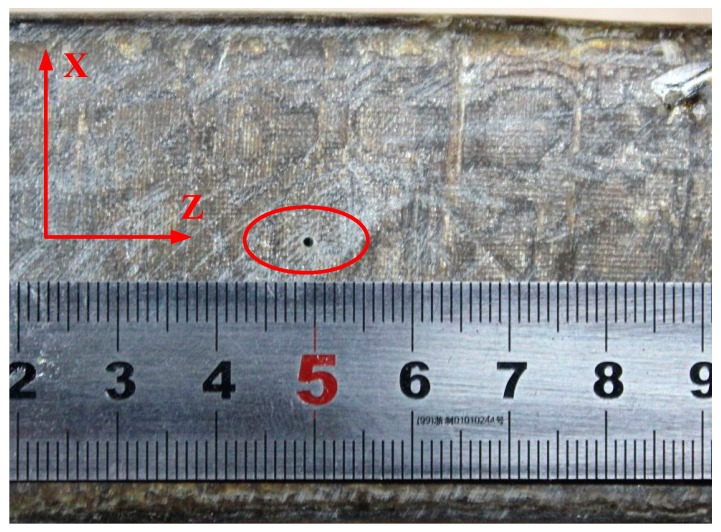
The longitudinal crack damage.

The stator bar specimens used in this experiment are taken from a large generator with a rate of 18 kV/300 MW. As shown in Figure 9, they are the 2.68 m slot bar, the 1.2 m slot bar and the 1.45 m end-winding bar, respectively. It is difficult to simulate the real stator insulation damage in experiments, and it is difficult to obtain enough samples of stator insulation damage. Therefore, based on typical damage model of stator insulation, it is an effective way to obtain typical damage samples of stator insulation by numerical simulation. In this study, 15 training samples were obtained by numerical simulation, and 5 damage testing samples were obtained by artificially manufacturing damage in stator bars in the experiment. Damage processing is as follows: It has been mentioned in Section 6 that three stator bars were used in the experiment. One surface crack damage is artificially produced on the 2.68 m slot bar (Figure 10). One longitudinal crack damage is artificially produced on the end-winding bar and three longitudinal crack damage of different size are artificially made on the 1.2 m slot bar (Figure 11). All three longitudinal crack damages are in the same position on the 1.2 m slot bar. In the experiment, a smaller one is manufactured firstly. The corresponding Lamb wave signals are obtained. Then the size of the damage is enlarged on the basis of the original damage, and the Lamb wave signals for another damage size are obtained and so on. 

Similar to the numerical simulation analysis, the features of received Lamb wave signals are extracted separately, and the type of insulation damage is identified by SVM classifier and ANN classifier. The damage identification results based on single damage feature and multi-feature fusion are shown in Table 5 and Table 6, respectively.

From Table 5 and Table 6, it can be seen that the accuracy of damage identification based on multi-feature is better than that of single feature. According to Table 6, the damage identification accuracy of testing samples increases with the number of training samples. Compared with the damage identification based on Lamb wave features, the insulation damage identification accuracy can be improved by predicting the damage geometry size of testing samples and fusing with Lamb wave features. In addition, the identification effect of SVM classifier is better than that of ANN classifier. The experimental results are similar to the simulation results. This paper mainly aims at single damage model to verify the validity and feasibility of multi-feature fusion and typical damage identification, which is also the basis of multi-damage identification. In practical application, there may be multiple damages to stator bar. The process of Lamb wave propagation in stator insulation structure is more complex because of reflection and scattering. Lamb wave detection wave packet can overlap, which will cause more obstacles to damage feature extraction, and multi-damage identification is more difficult. So, the identification of multiple damages needs further research.

## 7. Conclusions

In this paper, a damage identification method of stator insulation based on Lamb wave multi-feature fusion is proposed. From the simulation and experimental results, the following conclusions can be drawn:

The damage features of stator insulation extracted by HT, PSD, FFT, and WFD vary with the different size of damage. It indicates that the damage features extracted can effectively characterize the damage features of stator insulation, which lays a foundation for damage identification.

According to the stator insulation damage features, the type of stator insulation damage can be identified by SVM classifier. In addition, the classification effect of SVM is better than that of ANN classifier under the same conditions.

The accuracy of stator insulation damage identification using Lamb wave multi-feature fusion is higher than that of single feature. Furthermore, the insulation damage identification accuracy can be further improved by predicting the damage geometry size and fusing with Lamb wave features, which can provide effective reference information for stator insulation condition assessment of large generators.

## Figures and Tables

**Table 1:** Feature extraction results of different damage types.

Damage Size(mm)			WFD	HT (10^−10^ m)	PSD (10^−23^ W/Hz)	FFT (10^−7^ m)
x	y	z				
0	0	0	1.3641	3.101	1.702	1.397
20	1	2	1.56965	3.092	1.669	1.368
20	3	2	1.8877	3.081	1.666	1.367
20	5	2	2.1125	3.054	1.662	1.365
20	3	1	1.85395	3.084	1.667	1.368
20	3	2	1.8877	3.081	1.666	1.367
20	3	3	1.91445	3.078	1.663	1.366
10	3	2	1.7076	3.093	1.668	1.368
20	3	2	1.8877	3.081	1.666	1.367
30	3	2	1.99755	3.069	1.66	1.365
1	2	1	1.3739	3.101	1.67	1.368
3	2	3	1.45065	3.1	1.668	1.367
5	2	5	1.5441	3.098	1.665	1.365
1	4	1	1.38285	3.1	1.667	1.367
3	4	3	1.5179	3.099	1.666	1.366
5	4	5	1.66095	3.094	1.663	1.364
1	6	1	1.38925	3.099	1.666	1.365
3	6	3	1.5627	3.097	1.664	1.363
5	6	5	1.73155	3.09	1.66	1.36
5	1	5	1.7335	3.589	2.24	1.591
10	1	10	1.87065	3.577	2.13	1.561
15	1	15	2.2235	3.494	1.91	1.502
5	2	5	1.9889	3.563	2.133	1.558
10	2	10	2.0066	3.555	2.007	1.527
15	2	15	2.26645	3.483	1.897	1.489
5	3	5	2.07835	3.528	2.03	1.524
10	3	10	2.10295	3.475	1.945	1.494
15	3	15	2.29675	3.445	1.838	1.461
1	1	1	1.41915	3.601	2.29	1.605
2	2	2	1.95155	3.572	2.16	1.567
3	3	3	2.0727	3.54	2.06	1.534

**Table 2:** Damage identification results based on single feature.

Features	Number of Training Samples	Number of Testing Samples	Identification Results		Identification Accuracy	
			ANN	SVM	ANN	SVM
HT	16	13	2	3	15.4%	23.1%
FFT	16	13	3	4	23.1%	30.1%
PSD	16	13	5	6	38.5%	46.1%
WFD	16	13	3	5	23.1%	38.5%

**Table 3:** Evaluation results of damage size prediction values for 13 testing samples.

WFD	HT (10^−10^ m)	PSD (10^−^^23^ W/Hz)	FFT (10^−^^7^ m)	x	y	z	$\hat{x}$	$\hat{y}$	$\hat{z}$	$\left|\delta_{x}\right|$	$\left|\delta_{y}\right|$	$\left|\delta_{z}\right|$
1.56965	3.092	1.669	1.368	20	1	2	18.95	1.08	2.15	5.3%	8.0%	7.5%
1.91445	3.078	1.663	1.366	20	3	3	19.21	3.11	3.11	3.9%	3.7%	3.7%
1.85395	3.084	1.667	1.368	20	3	1	18.29	3.09	1.03	8.6%	3.0%	3.0%
1.7076	3.093	1.668	1.368	10	3	2	10.80	3.12	2.03	8.0%	4.0%	1.5%
1.45065	3.1	1.668	1.367	3	2	3	3.08	2.06	2.96	2.7%	3.0%	1.3%
1.5179	3.099	1.666	1.366	3	4	3	3.16	3.68	2.95	5.3%	8.0%	1.7%
1.66095	3.094	1.663	1.364	5	4	5	5.32	3.82	4.87	6.4%	4.5%	2.6%
1.5627	3.097	1.664	1.363	3	6	3	3.26	6.21	2.86	8.7%	3.5%	4.7%
1.87065	3.577	2.13	1.561	10	1	10	8.37	1.08	9.10	6.3%	8.0%	9.0%
2.26645	3.483	1.897	1.489	15	2	15	14.20	2.07	14.10	5.3%	3.5%	6.0%
2.0066	3.555	2.007	1.527	10	2	10	10.18	2.12	9.80	1.8%	6.0%	2.0%
2.1029	3.475	1.945	1.494	10	3	10	10.80	2.71	9.63	8.0%	9.7%	3.7%
1.95155	3.572	2.16	1.567	2	2	2	2.17	1.90	2.18	8.5%	5.0%	9.0%

**Table 4:** Damage identification results based on multi-feature fusion.

Number of Features	Number of Training Samples	Number of Testing Samples	Identification Results		Identification Accuracy	
			ANN	SVM	ANN	SVM
7 (HT, PSD, FFT, WFD, $\overset{\wedge }{x},\overset{\wedge }{y},\overset{\wedge }{z}$)	9	20	13	15	65%	75%
4 (HT, PSD, FFT, WFD)	9	20	12	13	60%	65%
7 (HT, PSD, FFT, WFD, $\overset{\wedge }{x},\overset{\wedge }{y},\overset{\wedge }{z}$)	13	16	12	14	75%	87.5%
4 (HT, PSD, FFT, WFD)	13	16	11	13	68.8%	81.2%
7 (HT, PSD, FFT, WFD, $\overset{\wedge }{x},\overset{\wedge }{y},\overset{\wedge }{z}$)	16	13	11	13	84.6%	100%
4 (HT, PSD, FFT, WFD)	16	13	10	12	76.9%	92.3%

**Table 5:** Experimental results of damage identification based on single feature.

Number of Features	Number of Training Samples	Number of Testing Samples	Identification Results		Identification Accuracy	
			ANN	SVM	ANN	SVM
HT	15	5	0	1	0.0%	20.0%
PSD	15	5	0	1	0.0%	20.0%
FFT	15	5	0	1	0.0%	20.0%
WFD	15	5	1	1	20.0%	20.0%

**Table 6:** Experimental results of damage identification based on multi-feature fusion.

Number of Features	Number of Training Samples	Number of Testing Samples	Identification Results		Identification Accuracy	
			ANN	SVM	ANN	SVM
7 (HT, PSD, FFT, WFD, $\overset{\wedge }{x},\overset{\wedge }{y},\overset{\wedge }{z}$)	8	5	1	3	20%	60%
4 (HT, PSD, FFT, WFD)	8	5	1	2	20%	40%
7 (HT, PSD, FFT, WFD, $\overset{\wedge }{x},\overset{\wedge }{y},\overset{\wedge }{z}$)	12	5	3	4	60%	80%
4 (HT, PSD, FFT, WFD)	12	5	2	3	40%	60%
7 (HT, PSD, FFT, WFD, $\overset{\wedge }{x},\overset{\wedge }{y},\overset{\wedge }{z}$)	15	5	3	5	60%	100%
4 (HT, PSD, FFT, WFD)	15	5	3	4	60%	80%
